# Perceived images and expected roles of Indonesian nurses

**DOI:** 10.1002/nop2.156

**Published:** 2018-05-17

**Authors:** Christine L. Sommers, Dame Elysabeth Tarihoran, Sandra Sembel, Huey‐Ming Tzeng

**Affiliations:** ^1^ Faculty of Nursing and Allied Health Sciences Universitas Pelitata Harapan Tangerang Indonesia; ^2^ College of Nursing University of Saskatchewan Saskatoon SK Canada

**Keywords:** expectation, image, Indonesia, nurse roles, nursing, perception

## Abstract

**Aim:**

The aim of this study was to explore how non‐nurses and nurses differ regarding the perceived images and expected roles of Indonesian nurses.

**Design:**

A cross‐sectional survey study

**Methods:**

An online tool shared via email was used to collect data in March 2014, from a convenient sample of 1,228 employees of a private university located in Karawaci, Indonesia. An English/Indonesian version of the survey was developed: 19 perception items and 19 expectation items using a 5‐point Likert scale. Independent sample *t* tests were used to compare groups.

**Results:**

One hundred and forty‐three people completed the survey; a response rate of 11.6%. Thirteen were nurses and 130 were non‐nurses. Compared with nurses, non‐nurses were less likely to agree with statements that Indonesian nurses are self‐sacrificing, provide help to others, are devoted to caring, perform housekeeping duties and are knowledgeable. Monitoring nurses' image on a regular basis is essential. A public education campaign could focus on selected positive characteristics to improve the image of Indonesian nurses.

## 1 INTRODUCTION

Indonesia is a transcontinental country located in Southeast Asia with some territories in Oceania. The ethnic groups include Chinese, Arab, Eurasian, Indian and Pakistani. About 85% of the population are Muslim (https://www.ncbi.nlm.nih.gov/mesh/?term=indonesia; accessed 6 April 2018). Indonesia is the fourth most populous country in the world, with more than 257 million people in 2015 (http://www.worldbank.org/en/country/indonesia; accessed 6 April 2018) and it has a high demand for healthcare services, primarily supported by nurses. Indonesian nurses have been perceived as physicians' helpers with a lower status than physicians. This public perception of nursing could discourage young people from considering nursing as a career (Shields & Hartati, 2003).

As a result, the purpose of this survey study was to explore the differences between individuals with a nursing background and those without this background in the perceived images and expected roles of Indonesian nurses. The main research question is: “What are the differences between nurses and non‐nurses in the perceived images and expected roles of Indonesian nurses?” This study is important in the recruitment of nurses into the profession.

## 2 THE PROBLEM

Birks, Chapman, and Francis (2009) claimed that from a global perspective, nursing could still be regarded as an oppressed profession. Nurses in Taiwan, a Chinese and Confucianism‐based society, are viewed as handmaidens to physicians, angels, individuals in immoral relationships and as uncaring and hardened individuals (Tzeng, 2006). Based on the results of the four developed ordinal logistic regression models, Tzeng (2006) concluded that six factors explained the strength of the levels of general perception towards the image of Taiwanese nurses. These six factors are: (a) being male; (b) having positive perceptions towards the angel of mercy, (c) the careerist aspects of nursing image; (d) having negative perceptions towards the bureaucratic aspect of the nursing image; (e) having less of a difference between the participants' perceptions and their expectations towards the romantic aspect of the nursing image; (f) being more satisfied with the professional services provided by Taiwanese nurses (Tzeng, 2006).

In a study conducted by Sollami, Caricati, and Mancini (2015) in Italy, nursing and medical students shared a stereotypical belief that compared with physicians, nurses were more communal, more socially competent and caring and medically less competent and less autonomous. Weaver, Salamonson, Koch, and Jackson (2013) studied 484 undergraduate nursing students at a large university in Australia and found that students were concerned that television promotes a negative image of the nursing profession. Such an image could hinder recruitment of the future nursing workforce and retention of those already in the nursing profession.

To develop an effective public education campaign to recruit and retain a nursing workforce, we need to understand the difference between nurses and non‐nurses in the perceptions and expectations of nurses. The current study repeated the study conducted in Taiwan by Tzeng (2006) and had a focus on exploring the image issue related to Indonesian nurses. The literature regarding the image of nursing in Indonesia appears to be limited to one study published in 2003 (Shields & Hartati, 2003).

## 3 METHODOLOGY

### 3.1 Design

A cross‐sectional, survey study used an online tool (SurveyMonkey^®^; http://www.surveymonkey.com) shared via email for data collection from active nursing and non‐nursing faculty and staff of a private university located in Karawaci, Indonesia. Data were collected in March 2014. The research review committee of the study university reviewed and approved the project.

### 3.2 Sample and procedures

The study used total sampling that included all faculty and staff with a university email address. There were no exclusion criteria for participation. Information about the study was delivered to the email addresses of all employees (*N* = 1,228). Individuals who were willing to participate in the study clicked on the online survey links provided in the email. Participation was voluntary and anonymous. The survey was open throughout March 2014. The online survey took approximately 15 min to complete. To encourage participation, participants were given the option to voluntarily enter their name and email address to be entered into a random drawing for a voucher to a local grocery store. Five random names were chosen. Each of the chosen participants received a voucher for 200,000 Indonesia Rupiah (about US$15).

### 3.3 Instrument

The tool used in this present study was based on a Chinese language version of a nurses' image survey tool (Tzeng, 2006), which was translated into English by the tool developer (a nurse researcher) and published in English in 2006. The English version was then translated into Indonesian by two of the nurse researchers who are fluent in both Indonesian and English. Each participant could choose to answer either the English or Indonesian version of the survey. The tool may be obtained from the corresponding author.

The appropriateness of the content and wording of the English/Indonesian version of the tool was validated by a five‐member expert panel of nurse educators, who are not the investigators of this study and who are fluent in English and Indonesian. The tool was modified, mostly for appropriate wording in Indonesian (e.g. selecting the correct Indonesian word/phrase for promiscuous). The author of the Chinese version tool also reviewed the English translation to ensure that the content and meaning were kept. The finalized tool was then tested on five non‐nurse working people they were not affiliated with the study university; their responses were not included in the final analysis. These participants did not have any suggestions for improving the tool and did not have any difficulty using SurveyMonkey to complete it.

Both the English and Indonesian versions of the tool have 19 items to assess participants' perception levels and another 19 items to assess their expectation levels (Table 1 for item statements). Each item was rated using a 5‐point Likert scale (five as very much agree or needed and one as very much disagree or not needed). As reported in Tzeng's (2006), the categorization of the nursing image items was determined based on factor analysis using the 19 expectation items for data reduction (Extraction method: Principal component analysis; Rotation method: Varimax with Kaiser Normalization; Eigenvalue is equal to larger than 1; *N* = 488). The five‐subscale solution was identified. These five subscales were named as the following dimensions: (a) angel of mercy (seven items); (b) romantic (four items); (c) careerist (four items); (d) obedient (two items); (e) bureaucratic (two items). The same five‐subscale item categorization was adopted in this Indonesia nurse study. The reliability Cronbach's alpha of the Chinese language tool was 0.85 for the perception items and 0.81 for the expectation items (Tzeng, 2006). The average values of the included items were calculated for each scale. In other words, based on Tzeng's (2006), the 19 perception items were grouped into five scales and the average value by each scale was calculated. The 19 expectation items were also grouped into five scales using the same item categorization and the average value by each scale was calculated. The tool also collected demographic characteristics and answers to four general questions.

**Table 1:** Participants' perceptions and expectations (*N *=* *143): Results of the independent‐sample *t* tests for equality

Perception scales^a^	Group	*N*	Mean (*SD*)^b^	*t*	*df*	Significance (two‐tailed)	Mean difference (nurses' rating—non‐nurses' rating)
P‐Scale 1. Angel of Mercy (the mean of the responses of items of 1, 2, 4, 6, 7, 8 and 9)	Nurses	13	3.55 (0.49)	0.48	141	.63	0.08
	Non‐nurses	130	3.47 (0.56)				
P‐Scale 2. Romantic (the mean of the responses of items 11, 12, 13, 14)	Nurses	13	2.67 (0.69)	0.55	141	.58	0.08
	Non‐nurses	130	2.59 (0.50)				
P‐Scale 3. Careerist (the mean of the responses of items 3, 15, 16 and 17)	Nurses	13	3.63 (0.92)	1.70	141	.09	0.34
	Non‐nurses	130	3.30 (0.66)				
P‐Scale 4. Obedient (the mean of the responses of items 5 and 10)	Nurses	13	3.46 (0.85)	1.37	141	.17	0.27
	Non‐nurses	130	3.20 (0.65)				
P‐Scale 5. Bureaucratic (the mean of the responses of items 18 and 19)	Nurses	13	3.50 (0.68)	0.47	141	.64	0.10
	Non‐nurses	130	3.40 (0.74)				
Expectation scales^a^							
E‐Scale 1. Angel of Mercy (the mean of the responses of items of 1, 2, 4, 6, 7, 8 and 9)	Nurses	13	4.05 (0.33)	−1.52	141	.13	−0.20
	Non‐nurses	130	4.25 (0.46)				
E‐Scale 2. Romantic (the mean of the responses of items 11, 12, 13, 14)	Nurses	13	2.94 (0.91)	1.37	141	.17	0.33
	Non‐nurses	130	2.62 (0.81)				
E‐Scale 3. Careerist (the mean of the responses of items 3, 15, 16 and 17)	Nurses	13	4.69 (0.59)	1.55	141	.12	0.24
	Non‐nurses	130	4.45 (0.52)				
E‐Scale 4. Obedient (the mean of the responses of items 5 and 10)	Nurses	13	3.04 (0.69)	−0.90	141	.37	−0.24
	Non‐nurses	130	3.28 (0.95)				
E‐Scale 5. Bureaucratic (the mean of the responses of items 18 and 19)	Nurses	13	4.23 (1.05)	0.46	141	.64	0.09
	Non‐nurses	130	4.14 (0.64)				
Perception items^a^“Based on your perception of the Indonesian nurses' image, please indicate for each statement how much you agree or disagree.”							
P1. Indonesian nurses are self‐sacrificing	Nurses	13	3.77 (0.93)	2.03	141	.04*	0.55
	Non‐nurses	130	3.22 (0.93)				
P2. Indonesian nurses are ethical	Nurses	13	3.92 (0.86)	1.92	141	.06	0.45
	Non‐nurses	130	3.47 (0.81)				
P3. Indonesian nurses are honourable	Nurses	13	3.62 (1.04)	0.68	141	.50	0.16
	Non‐nurses	130	3.45 (0.79)				
P4. Indonesian nurses provide help to others	Nurses	13	4.31 (0.63)	2.75	141	<.01**	0.64
	Non‐nurses	130	3.67 (0.81)				
P5. Indonesian nurses obey without questioning	Nurses	13	3.38 (1.04)	1.08	141	.28	0.28
	Non‐nurses	130	3.10 (0.89)				
P6. Indonesian nurses are assistants to physicians	Nurses	13	1.69 (1.18)	−7.11	141	<.01**	−1.99
	Non‐nurses	130	3.68 (0.94)				
P7. Indonesian nurses are brave/courageous	Nurses	13	3.38 (0.96)	0.13	141	.90	0.03
	Non‐nurses	130	3.35 (0.81)				
P8. Indonesian nurses are devoted to caring	Nurses	13	4.08 (0.76)	2.13	141	.03*	0.53
	Non‐nurses	130	3.55 (0.86)				
P9. Indonesian nurses are motherly	Nurses	13	3.69 (0.95)	1.37	141	.17	0.33
	Non‐nurses	130	3.36 (0.82)				
P10. Indonesian nurses are obedient/passive	Nurses	13	3.54 (1.13)	0.99	141	.32	0.25
	Non‐nurses	130	3.29 (0.82)				
P11. Indonesian nurses are committed to housekeeping duties	Nurses	13	3.77 (0.83)	2.34	141	.02*	0.50
	Non‐nurses	130	3.27 (0.72)				
P12. Indonesian nurses are sensual	Nurses	13	2.31 (1.25)	−0.06	141	.97	−0.02
	Non‐nurses	130	2.32 (0.83)				
P13. Indonesian nurses are romantic.	Nurses	13	2.62 (0.96)	1.20	141	.23	0.27
	Non‐nurses	130	2.35 (0.75)				
P14. Indonesian nurses are promiscuous/immoral	Nurses	13	2.00 (0.91)	−1.70	141	.09	−0.42
	Non‐nurses	130	2.42 (0.85)				
P15. Indonesian nurses are intelligent/analytical	Nurses	13	3.54 (1.05)	1.47	141	.14	0.35
	Non‐nurses	130	3.18 (0.81)				
P16. Indonesian nurses are knowledgeable	Nurses	13	3.69 (0.95)	2.98	141	<.01**	0.68
	Non‐nurses	130	3.01 (0.77)				
P17. Indonesian nurses are respected professionals	Nurses	13	3.69 (1.03)	0.58	141	.56	0.15
	Non‐nurses	130	3.54 (0.90)				
P18. Indonesian nurses do everything asked and everything necessary	Nurses	13	3.85 (0.69)	1.80	141	.07	0.43
	Non‐nurses	130	3.42 (0.83)				
P19. Indonesian nurses pay attention to their working organizations' structure and clinical ladder career path	Nurses	13	3.15 (1.14)	−0.90	141	.37	−0.23
	Non‐nurses	130	3.38 (0.86)				
Expectation items^a^“Based on your expectation of the Indonesian nurses' image, please indicate for each statement how much you agree or disagree.”							
E1. An Indonesian nurse should be self‐sacrificing	Nurses	13	4.31 (0.48)	0.12	141	.90	0.02
	Non‐nurses	130	4.28 (0.66)				
E2. An Indonesian nurse should be ethical	Nurses	13	4.62 (0.51)	0.43	141	.67	0.07
	Non‐nurses	130	4.55 (0.56)				
E3. An Indonesian nurse should be honourable	Nurses	13	4.69 (0.48)	1.55	141	.12	0.27
	Non‐nurses	130	4.42 (0.61)				
E4. An Indonesian nurse should provide help to others	Nurses	13	4.69 (0.48)	0.83	141	.41	0.14
	Non‐nurses	130	4.55 (0.59)				
E5. An Indonesian nurse should obey without questioning	Nurses	13	3.46 (1.05)	−0.12	141	.90	−0.04
	Non‐nurses	130	3.50 (1.11)				
E6. An Indonesian nurse should be an assistant to physicians	Nurses	13	1.85 (0.99)	−5.70	141	<.01**	−1.74
	Non‐nurses	130	3.59 (1.06)				
E7. An Indonesian nurse should be brave/courageous	Nurses	13	4.15 (0.56)	−0.37	141	.71	−0.07
	Non‐nurses	130	4.22 (0.65)				
E8. An Indonesian nurse should be devoted to caring	Nurses	13	4.69 (0.48)	0.96	141	.34	0.15
	Non‐nurses	130	4.54 (0.56)				
E9. Indonesian nurse should be motherly	Nurses	13	4.08 (1.04)	0.11	141	.92	0.02
	Non‐nurses	130	4.05 (0.72)				
E10. An Indonesian nurse should be obedient/passive	Nurses	13	2.62 (1.19)	−1.36	141	.18	−0.45
	Non‐nurses	130	3.06 (1.13)				
E11. An Indonesian nurse should be committed to housekeeping duties	Nurses	13	4.08 (0.86)	1.24	141	.22	0.32
	Non‐nurses	130	3.75 (0.90)				
E12. An Indonesian nurse should be sensual	Nurses	13	2.38 (1.39)	0.60	141	.56	0.24
	Non‐nurses	130	2.15 (1.05)				
E13. An Indonesian nurse should be romantic	Nurses	13	2.77 (1.30)	1.84	141	.07	0.54
	Non‐nurses	130	2.23 (0.98)				
E14. An Indonesian nurse should be promiscuous/immoral	Nurses	13	2.50 (1.76)	0.42	141	.68	0.20
	Non‐nurses	130	2.34 (1.64)				
E15. An Indonesian nurse should be intelligent/analytical	Nurses	13	4.92 (0.28)	4.53	141	<0.01**	0.43
	Non‐nurses	130	4.49 (0.64)				
E16. An Indonesian nurse should be knowledgeable	Nurses	13	4.62 (1.12)	0.80	141	.42	0.15
	Non‐nurses	130	4.46 (0.60)				
E17. An Indonesian nurse should be a respected professional	Nurses	13	4.54 (1.13)	0.51	141	.61	0.10
	Non‐nurses	130	4.44 (0.62)				
E18. An Indonesian nurse should do everything asked and everything necessary	Nurses	13	3.92 (1.19)	−0.56	141	.58	−0.14
	Non‐nurses	130	4.06 (0.81)				
E19. An Indonesian nurse should pay attention to his or her working organization's structure and clinical ladder career path	Nurses	13	4.54 (1.13)	1.56	141	.12	0.32
	Non‐nurses	130	4.22 (0.66)				

a Both the English and Indonesian versions of the tool have 19 items to assess participants' perception levels using a 5‐point Likert scale (1 = very much disagree, 2 = disagree, 3 = neutral, 4 = agree, 5 = very much agree) and another 19 items to assess expectation levels, using a different 5‐point Likert scale (1 = very little needed, 2 = little needed, 3 = neutral, 4 = needed, 5 = very much needed). Participants were asked: “Based on your perception of the Indonesian nurses' image, please indicate for each statement how much you agree or disagree.” and “Based on your expectation of the Indonesian nurses' image, please indicate for each statement how much you agree or disagree.” The average values of the included items were calculated for each scale. The 19 perception items were grouped into five scales and the average value by each scale was calculated. The 19 expectation items were also grouped into five scales using the same item categorization and the average value by each scale was calculated.

b The higher values of the group means are underlined for easy reference.

*p < .05, two‐tailed.

**p < .01, two‐tailed.

### 3.4 Data analysis

Only fully completed surveys were included in the analysis. Data were analysed using SPSS Version 21 (IBM Corp., Armonk, NY, USA). The independent sample *t* test was used to detect the response differences between the nurse and non‐nurse groups on the computed scales and individual items. The level of significance was set at .05.

## 4 RESULTS

A total of 174 participants responded to the survey; 143 participants filled out the survey completely, yielding a response rate of 11.6%. Thirteen (9.1%) had nursing backgrounds and 130 (90.9%) had no nursing backgrounds. The average age was 33.9 years (*SD* 10.3, range of 15–75) and most were female (65.7%, *N* = 94). The education levels were doctoral (8.4%, *N* = 12), master (38.5%, *N* = 55), bachelor (46.9%, *N* = 67) and less than bachelor (6.3%, *N* = 9). Most participants were Chinese (45.5%, *N* = 65), followed by Javanese (21.7%, *N* = 31), Bataknese (21%, *N* = 30), Sundanese (1.4%, *N* = 2) and other (10.48%, *N* = 15). The reliability Cronbach's alpha of the English/Indonesian version of the tool for the 19 perception items was .84 and for the 19 expectation items was .82 (*N* = 143).

As shown in Table 1, no difference was found between nurses and non‐nurses in the levels of the five perception scales and the five expectation scales with *p* value > .05. For the 19 perception items, the non‐nursing participants were statistically significantly less likely to agree on the items of Indonesian nurses being self‐sacrificing (item 1), providing help to others (item 4), being devoted to caring (item 8), being committed to housekeeping duties (item 11) and being knowledgeable (item 16) compared with the nursing participants. The non‐nursing participants were statistically significantly more likely to agree on the item of Indonesian nurses being assistants to physicians (item 6) than nursing participants. For the 19 expectation items, the non‐nursing participants had statistically significant higher expectation levels for Indonesian nurses being assistants to physicians (item 6) and being intelligent and analytical (item 15) than nursing group participants.

## 5 CONCLUSIONS

The research question of what the differences between nurses and non‐nurses in the perceived images and expected roles of Indonesian nurses are answered. No difference was found between nurses and non‐nurses in the levels of the five perception scales and the five expectation scales. On the individual perception and expectation items, nurses and non‐nurses differed with regard to six perceived images and two expected roles of Indonesian nurses. Compared with nurses, non‐nurses were less likely to agree with the perception statements that Indonesian nurses are self‐sacrificing, provide help to others, are devoted to caring, are committed to housekeeping duties and are knowledgeable. Thus, the non‐nursing participants were more likely to agree with the perception statement that Indonesian nurses are assistants to physicians. In contrast, non‐nurses were more likely than nurses to expect Indonesian nurses to be assistants to physicians and to be intelligent and analytical. These findings are similar to the study conducted by Shields and Hartati (2003) in Indonesia and the one conducted by Tzeng, 2006; in Taiwan.

As for study limitations, due to the differences in the study designs, comparison of the findings of the present study with the ones conducted by Shields and Hartati (2003) and Tzeng (2006) should be cautious. Generalization of this cross‐sectional study's finding should be careful as this study's population was from only one single private university in Indonesia, as a study limitation. Another study limitation is the low response rate (11.6%) with only 13 nurses participated in this study (one‐tenth of the 130 non‐nurse participants).

As for future research, longitudinal, multi‐region and multi‐nation comparison studies are warranted. Future studies may include a more diverse sample and use mixed method study designs to explore the optimum public education campaign themes for promoting nurses' image. As for practical implications, to minimizing the gap between non‐nurses' and nurses' expectation and perception levels of nursing services, nursing educators and leaders should continue to monitor patients' and potential future customers' perceptions towards the image of nurses in Indonesia and work to improve that image, such as through on‐job training to strengthen nurses' caring attitudes and communication skills to patients and their family members. To improve nurses' professional image, it is essential to monitor nurses' image via the public eyes as well as nurses' self‐assessment on a regular basis. A public education campaign to improve the image of Indonesian nurses could focus on selected positive characteristics, such as Indonesian nurses being devoted to caring, providing help to others and being knowledgeable.

## CONFLICT OF INTEREST

No conflict of interest has been declared by the authors.

## AUTHOR CONTRIBUTIONS

CLS, Ns. DET, SS and HMT were involved in the research design, data collection and analysis and manuscript preparation.
